# Supplementary figures and images for: The histone H3K4 demethylase JARID1A directly interacts with haematopoietic transcription factor GATA1 in erythroid cells through its second PHD domain
Source: R Soc Open Sci. 2020 Jan 29;7(1):191048. doi: 10.1098/rsos.191048 (PMC7029945; doi:10.1098/rsos.191048)

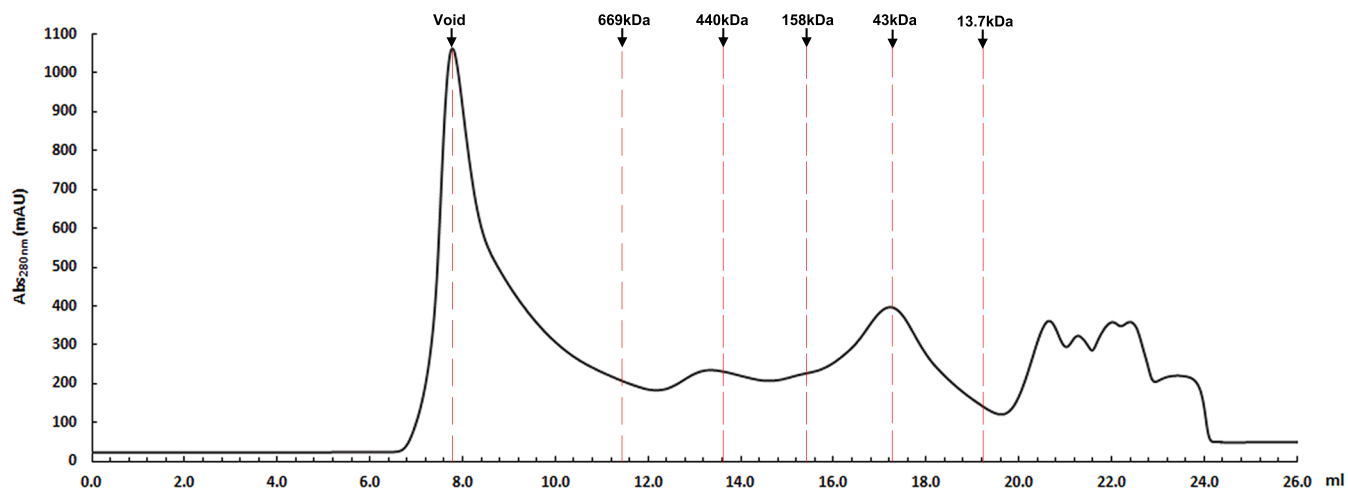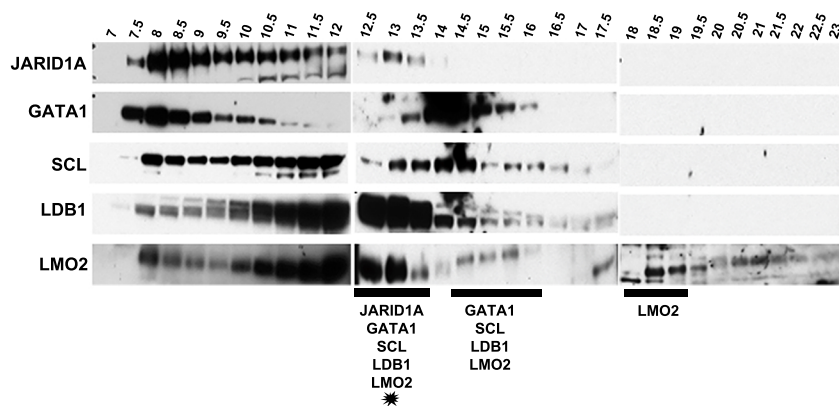

**Supplementary Fig. 1**

Supplement: Figure S1 [file rsos191048supp1.pdf]

# 500 MHz $^1\text{H}$ - $^{15}\text{N}$ HSQC spectrum of JARID1A PH2

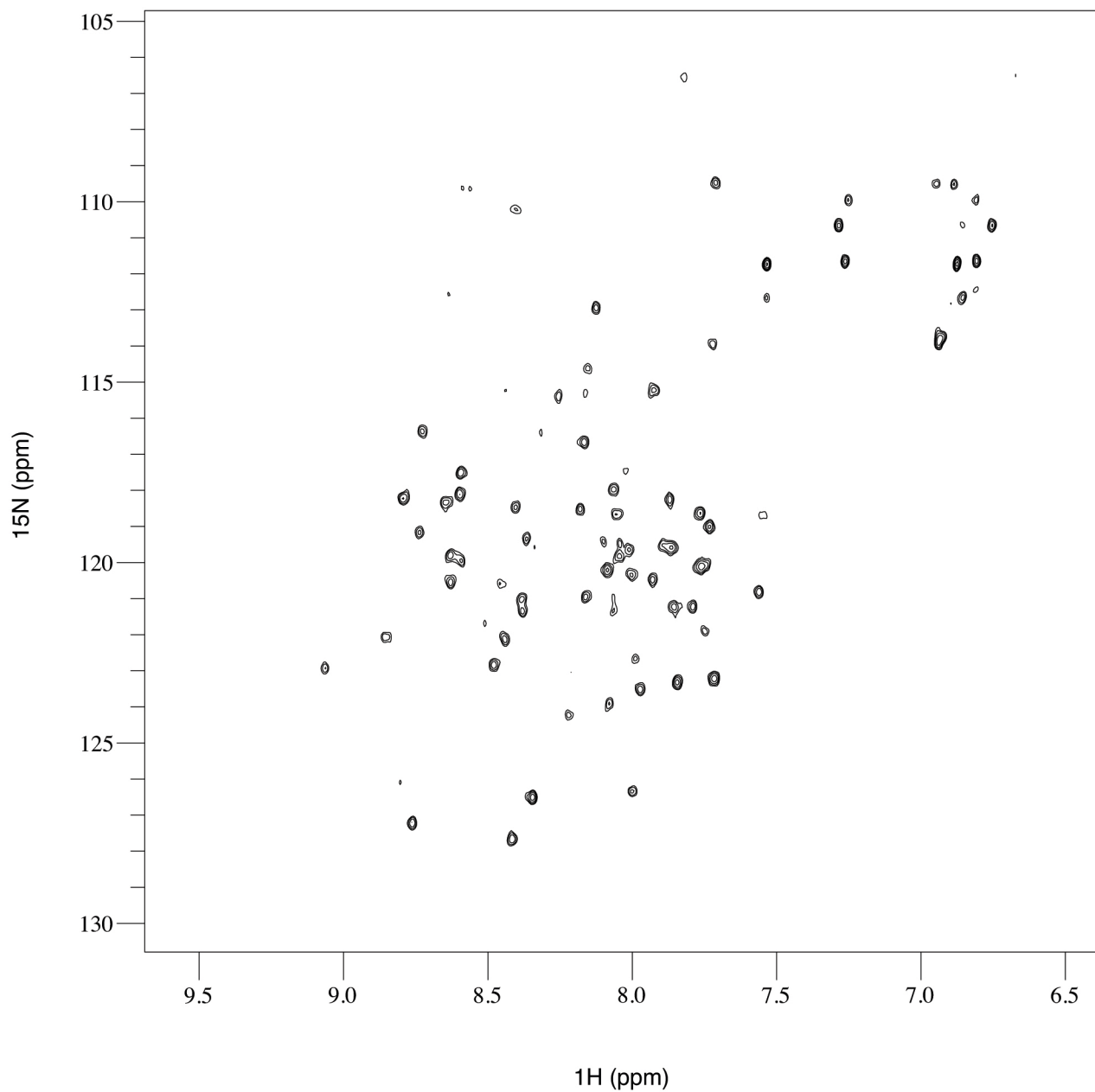

**Supplementary Fig. 2**

Supplement: Figure S2 [file rsos191048supp2.pdf]
