# Supplementary material for: The histone H3K4 demethylase JARID1A directly interacts with haematopoietic transcription factor GATA1 in erythroid cells through its second PHD domain
Source: R Soc Open Sci. 2020 Jan 29;7(1):191048. doi: 10.1098/rsos.191048 (PMC7029945; doi:10.1098/rsos.191048)
Supplement: Supplementary Figure captions [file rsos191048supp3.docx]

**Supplementary Figure1. Co-fractionation of JARID1A with the SCL-GATA1 complex by size exclusion chromatography.** Nuclear extracts from MEL cells were fractionated using Superose 6 size exclusion column. The elution fractions were analysed by Western blotting Western blotting analysis was performed using NuPAGE precast gels (3-8% Tris-acetate for JARID1A and 4-12% Bis-tris for SCL, GATA1, LMO2 and LDB1; Life Technologies) according to manufacturer’s instructions. Primary antibodies used were: JARID1A (ab70892; Abcam), SCL (sc-12984; Santa Cruz), LDB1 (sc-11198; Santa Cruz), LMO2 (MCA2744GA, ABD serotec) and GATA1 (sc-1234; Santa Cruz). Chromatography profile and western blot analysis are shown. The elution positions of the molecular weight standards (669, 440, 158, 43, 13.7 kDa) and that of the void volume are indicated above the chromatogram.

**Supplementary Figure2. 500 MHz ^1^H–^15^N HSQC spectrum of JARID1A PH2.** ^15^N-single-labelled His-Tag PHD2 was grown in M9 minimal media containing ^15^N–NH_4_Cl (1 g/L) (Sigma-Aldrich) as sole nitrogen source and purified as described in the Materials and Methods section. ^15^N -labelled sample of His-Tag PHD2 was probed in 20 mM NaH2PO4 pH 8.0 (95% H_2_O/ 5% D_2_O (v/v)), 100 mM NaCl at a concentration of 57.9 μM. NMR experiments were carried out at 293 K using a 500 MHz spectrometer equipped with Bruker Avance console and TCI CryoProbe.
